# An analysis of online health information on schizophrenia or related conditions: a cross-sectional survey

**DOI:** 10.1186/1472-6947-13-98

**Published:** 2013-08-30

**Authors:** Christina Athanasopoulou, Heli Hätönen, Sanna Suni, Christos Lionis, Kathleen M Griffiths, Maritta Välimäki

**Affiliations:** 1 Department of Nursing Science, University of Turku, Turku, Finland; 2 Clinic of Social and Family Medicine, School of Medicine, University of Crete, Heraklion, Greece; 3 Centre for Mental Health Research, The Australian National University, Canberra, ACT, Australia; 4 Southwest Hospital District, Turku, Finland

**Keywords:** Consumer health information, Information retrieval, Internet, Schizophrenia

## Abstract

**Background:**

Around 20% of those who seek health information online, search specifically for mental health. However, little is known about the nature of the online health information offered by two European countries, Finland and Greece, which are characterized by markedly differing levels of Internet access and online health information seeking. This study aims to assess, describe and compare websites, written in two European, non-English languages (Finnish and Greek) that appear first after performing an online search concerning schizophrenia or related conditions.

**Methods:**

The first 20 results from four search terms (searched in Finnish and Greek) in the Web search engine ‘Google’ were screened. A total of 160 websites were retrieved (80 Finnish, 80 Greek) and evaluated using a preformulated coding system which consisted of websites’ indicators, such as: types, characteristics, accountability, interactivity, aesthetics and content. Differences between websites were evaluated with Chi-Square or Fisher’s Exact tests for categorical data and independent t-tests for parametric data.

**Results:**

Twenty-four Finnish and thirty-four Greek websites (36% in total) were included. Almost two-thirds (62%, n=36) were owned by an organization, compared to 17% (n=10) by an individual. In both countries, aesthetics had the highest score (possible range 0–4, mean = 2.6, SD = .62), while interactivity the lowest (range 0–5, mean = 1.79, SD = .87). There were no statistically significant differences among the accountability, interactivity and aesthetics scores of the Finnish and Greek websites.

**Conclusions:**

All assessed indicators suggest there is a need to improve Finnish and Greek online information about schizophrenia or related conditions. The poor website interactivity is of particular concern given the challenges faced by the target group. The findings can be used to guide the development and dissemination of online mental health information aimed at Finnish and Greek online health-seekers.

## Background

Schizophrenia is a chronic, severe, debilitating mental disorder [1,2], which ranks third in global burden among the mental disorders [3]. Approximately 25 million people are diagnosed with schizophrenia globally [4], about 2.5 million in the USA [5] and around 3.7 million in Europe [6]. Schizophrenia affects the lives of those with the disorder and their caregivers [7,8] due to hallucinations, perceptual and communication difficulties [9]. The illness itself is also connected with high levels of fear, shame, guilt [10], and social isolation [7], which may lead to an unwillingness to adhere to treatment [11], consequent relapse [12] and readmissions [8,13].

A Cochrane review [14] concluded that psychoeducation may promote adherence to medication, diminish relapses, reduce the length of hospitalization and rates of readmission for those with schizophrenia. Advances in technology allow psychoeducation to be delivered conveniently through the Internet [15]. Such technology is now frequently used by members of the public to access health information online [16] with a substantial percentage of all online health searches being for mental health information [17]. Internet users visit medical websites primarily to acquire information about a health condition, its treatment, symptoms, and to obtain health advice [18].

Recent evidence suggests that the Internet is also an important source of illness-related information for people with schizophrenia [19] who value the availability of reliable, evidence-base information on the Internet [19] and the role it can play in increasing their sense of empowerment by increasing their understanding of their illness and capacity to communicate with their doctors [19]. In addition, web-based psychoeducation for people with schizophrenia shows some promise as a means of improving mental state, providing social support, and supporting medication compliance [20].

Schizophrenia is commonly associated with deficits in attention, concentration, visual perception and interpretation [1,9]. At the same time, online health information can be easily misinterpreted [21] and online health seekers may be exposed to contradictory medical advice and opinions [22]. As a consequence, it is particularly important that websites providing information about schizophrenia should present simple but high quality and understandable information, in a design format that takes into account the above difficulties [23,24].

The Internet is a potentially useful tool for retrieving information about mental illness [25,26]. In the USA, it is estimated that 21% of information health-seekers use the Internet for mental health issues [17]. In the UK, it has been reported that about 18% of all Internet users and 31.5% of Internet users with a past history of mental health problems used the Internet for retrieving mental health information [27]. However, patterns of computer and Internet use vary substantially across countries [28]. Two-thirds of the total population in the European Union (EU) use the Internet at least once a week [29], but there are marked discrepancies in Internet usage between member states of the EU. For example, in Southern Europe and specifically in Greece, 50% of households have Internet access, while the percentage in a Northern European country, such as Finland, is 84% [29]. Further, in 2011, 58% of Finnish citizens used the Internet to search for health-related information (second after Icelanders), whereas Greek citizens (30%) are among the least likely of the European Union members (after Italy, Cyprus and Romania) to use the Internet for health information seeking [30]. While a basic cornerstone of the European agenda is the right of freedom of movement across the EU [31], support of homogeneous rights, quality health services for citizens [32], and intercountry collaboration [33], priority should be given ensuring the availability and universal accessibility of high quality online mental health information [34-36]. The quality of the provided online health or mental health information, worldwide and specifically in Europe [37,38] still remains unclear. To our knowledge, four studies of the quality of online health information have been conducted in Finland [39,40], and Greece [41,42]. Only one of these was related to mental health. It found that Finnish websites providing information about antidepressants did not cover all aspects of treatment [39]. These two European countries differ in population, Internet access and, Information and Communication Technologies (ICT) use and attitudes [28-30]. The objective of the current study was to assess, describe and compare Finnish and Greek websites that first appear after performing an online search concerning schizophrenia or related condition.

## Methods

### Data collection and sample

On November 30, 2011, the Web search engine ‘Google’ was used to identify online health information on schizophrenia or related conditions. Google was selected because it is the most frequently used search engine [43] and the one of the most likely to be used by someone searching for online health information. Country specific versions for Finland (http://www.google.fi/) and Greece (http://www.google.gr/) were employed.

Search terms in Finnish and Greek language were chosen with the aim of generating a list of websites that might be similar to a search produced by a Finnish or Greek adult with average medical, Internet, and computer expertise [34,44,45]. Since searches are often triggered by a particular diagnosis or condition [17,46], we hypothesized that the four most probable search terms for someone within the spectrum of schizophrenic disorders would be: mental illness (‘mielisairaus’/‘ψυχική ασθένϵια’), mental disorder (’mielenterveyden häiriö’/‘ψυχική διαταραχή’), schizophrenia (‘skitsofrenia’, ‘σχιζφφρένϵια’), and psychosis (‘psykoosi’/‘ψύχωση’) respectively in Finnish and in Greek language.

The first 20 websites returned by the search engine for each of the four search terms were examined for eligibility (N = 160; 80 Finnish and 80 Greek). It was considered unlikely that the typical consumer would click on more than 20 results [47-49] from a single search. The 20 direct links from each ‘Google’ search term were saved. Screenshots of the direct webpages appearing from every ‘Google’ result were taken.

Additionally, within every website five webpages were collected, applying convenience sampling. After opening and saving the first webpage that appeared from Google results, then four additional randomly selected webpages were saved which were linked to the initial page. Convenience sampling was preferred for the selection of webpages [50] because of their convenient accessibility and proximity.

To be included in the study, a valid website was required to satisfy the following criteria: 1) focused on health or mental health issues for adults in the Finnish or Greek language; and 2) was a standard information website, open web-based encyclopaedia (e.g. ‘wikipedia’), discussion forum, blog or wiki. The latter was included since it has been reported that they are favoured online sources among people with schizophrenia [19]. Websites were excluded if they: 1) were not focused on health or mental health issues; 2) targeted educators or special schools’ educators or described courses; 3) primarily involved advertisements or book promotion; 4) were links leading to external files (e.g. .doc, .pdf, .ppt); 5) were invalid addresses or malware; 6) were incidental mental health articles or discussion in a non-health oriented forum or blog; 7) were not written in the Finnish or Greek languages; 8) or provided health information for a non-adult population (e.g. for children or adolescents, or their parents).

**Figure 1:**
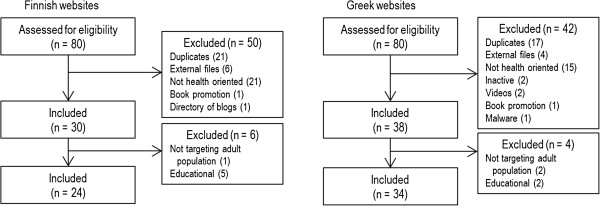
Flow diagrams of included websites.

A total of 56 Finnish websites and 46 Greek websites failed to satisfy the inclusion criteria and were excluded (Figure 1). This left 24 Finnish and 34 Greek language websites (58 sites in total) for analysis.

### Coding system and instrument

The coding system used for the data categorization consisted of six indicators: 1) type of website; 2) characteristics; 3) accountability; 4) interactivity; 5) aesthetics; and 6) content.

First, websites were categorized in seven types according to an adaptation of Morel et al. [51] (Table 1). Second, characteristics of the sites were evaluated according to criteria used by Griffiths & Christensen [34,45]. Three additional characteristics were added including other languages available, provision of online services (e.g. video-conferencing), and presence of the Health On the Net (HON) code [52] which is an ethical standard certification of the trustworthiness of a specific health and medical website (Table 1). Third, accountability was evaluated with the adapted version of Silberg et al’s [53] scale [45] (Table 1). The maximum total score of this scale is 9 (possible range 0–9). Fourth, interactivity was evaluated with the adapted version of Khazaal et al. [54,55] (Table 1). The maximum score on this scale is 5 (range 0–5). Fifth, aesthetics was evaluated with the adapted version of Kisely et al.’s criteria [56] originally introduced by Abbott [55] (Table 1). The maximum score on this scale is 4 (range 0–4).

**Table 1:** Coding tool for Finnish and Greek websites

**Indicators**	**Items**
**Types**^**a**^	Commercial
(only one possible option)	Personal page dev. by professional(s)
	University website
	Non-profit organization
	Governmental
	Open source
	Other (e.g. blogs)
**Characteristics**^**a**^	Scope:
(yes=1, no=0)	Specific (mental health)
No total score	Broad (health)
	Broader (general information)
	Ownership structure:
	Individual
	Organization
	Unknown
	Ownership type:
	Commercial
	Professional
	Consumer
	Unknown
	Country of origin:
	Finland
	Greece
	EU
	Other
	Drug company involved
	Professional Editorial board involved
	Health professional involved
	Promotion of prod/service
	Disclaimer
	Other languages available
	Online services
	HON certification
**Accountability**	Authorship^b^:
(yes=1, no=0)	Credited
(score 0–9)	Affiliations
	Credentials
	Attribution^b^:
	Source given
	Reference given
	Disclosure^a^:
	Site ownership
	Site sponsorship
	Currency^a^:
	Modified last 1/12 months
	Last date of mod. specified
**Interactivity**^**a**^	Intra-site search engine
(yes=1, no=0)	Audio/Video support
(score 0–5)	Evaluation quest.
	Supportive bodies
	Possibility to contact webmaster
**Aesthetics**^**b**^	Headings/subheadings
(yes=1, no=0)	Diagrams
(score 0–4)	Hyperlinks
	Absence of ads
**Content**^**c**^	Diagnosis
(yes=1, no=0)	Treatment
No total score	Patient association information
	Clinics information

^a^Navigation through website.

^b^Five random webpages within website.

^c^Direct webpage.

Lastly, the content of the provided online information was evaluated based on the availability of answers to health-related enquiries frequently generated by people with psychiatric disorders, such as information about diagnosis, treatment options, and medication side-effects [54]. Answers were searched using the screenshots of the direct webpages and not through website navigation. This decision was made because online health-seekers do not spend a lot of time searching for information within a website [47,57], and people with schizophrenia face difficulties with website navigation [23,24]. The searched items were: 1) diagnosis; 2) treatment; 3) information about patient associations; and 4) information about clinics (Table 1). If a webpage did not specifically state ‘symptom’ or ‘diagnosis’ of schizophrenia or related mental disorders, the page was considered not to provide information about symptoms or diagnosis, although these may have incorporated an obscure reference to this information. In addition, if the term ‘treatment’ or ‘diagnosis’ was mentioned (e.g. ‘The quicker the psychosis is diagnosed and the treatment is started, the better the recovery’) but a specific treatment option was not mentioned (e.g. ‘The treatment consists of medication and psychotherapy’), then it was not considered to contain treatment information. This decision was taken because people with schizophrenia may require concrete descriptions [23] to understand the text they are reading.

Coding was conducted as follows. First, researchers independently familiarized themselves with the retrieved websites. Second, they undertook an evaluation of website type, characteristics, accountability, interactivity, aesthetics, (CA for Greek and SS for Finnish data) and content of the webpages (CA and CB for Greek and MV and HH for Finnish data), with each website score on each of the criteria with ‘1’ indicating the criterion was satisfied and ‘0’ that it was absent. In addition, type, characteristics, disclosure, currency, interactivity were determined through navigation within the website, while authorship, attribution, and aesthetics were scored according to 5 random webpages within a single website. This approach was adopted, since in many cases, a single website was diverse (e.g. name of author was mentioned on one page but not the author of a text on another page). Content was evaluated only through the direct webpage retrieved from the ‘Google’ search, with the aim of evaluating that part of the website most health-seekers were likely to access (Table 1).

Reliability of the coding was ensured by following actions. First, two raters (CA, SS) critically examined the Greek and Finnish ratings concerning website type, characteristics, accountability, interactivity and aesthetics. The evaluators discussed any discrepancies in the decisions made with the final rating made by agreement. Reliability was investigated by recoding (third independent rater) a random selection of 20% of the websites. The number of scoring errors (n = 65) was then divided by the number of coded cells (N = 2538), yielding an error rate of 2.56%. Second, inter-rater reliability was assessed separately for the Finnish and Greek analysis. The percent agreement was calculated by dividing the number of observations in which the raters agreed by the total observations (97%). Third, any scoring disagreements in content analysis were resolved through discussion between the two same-language raters. Percent agreement for content analysis was 80% and 83% for the Finnish and Greek data respectively [58].

### Data analysis

Accountability, interactivity, and aesthetics indicator scores were computed by summing the value of each score of the item (yes = 1; no = 0). Correlations among accountability, interactivity and aesthetics were analyzed using Pearson’s correlation coefficient. Differences between categorical variables was evaluated using Chi-Square tests or Fisher’s Exact test if expected cell counts were <5. Differences in indicator scores between Finnish and Greek websites were analysed using independent t-tests. SPSS version 19.0 (IBM Corporation, Somers, NY, USA) was used for all statistical analyses and in all tests, an alpha of .05 was employed for identifying a statistically significant difference.

## Results

### Sample

The 58 included websites were: commercial (n = 18), personal pages developed by health professionals (n = 7), university websites (n = 1), non-profit organizations (n = 7), governmental (n = 2), open source (n = 5) websites where anyone could modify the webpage content, for example ‘wikis’ or online encyclopedias, or other sources (n = 18), for example, blogs or patient/professional associations.

### Characteristics of the websites

Fifty-five of the websites originated from Finland or Greece (24 Finnish, 31 Greek) and three were maintained by the European Commission. About one third (34%, n = 11) of Finnish and Greek (29%, n = 7) websites provided other language options, typically, English and/or Swedish in Finland and, English and/or French in Greece. Online services, e.g. video-conferencing or web telephony counseling were provided by 6% of Greek websites (n = 2), but not by Finnish websites. Three Greek websites but none of the Finnish websites had the HON certification.

Comparing Finnish and Greek website characteristics, there were statistically significant differences for three factors. First, a greater percentage of the Greek than Finnish websites were owned by a health professional (56% vs. 21%, *p* = .008). Second, there was a higher probability that a Greek website involved a health professional in the content development (76% vs. 38%, *p* = .003). Third, Greek websites promoted products or services more often than their Finnish counterparts (50% vs. 21%, *p* = .024) (Table 2).

**Table 2:** Characteristics of Finnish and Greek websites

**Characteristics**	**Total**	**FI**	**GR**	**Chi-square**	***P***^**a**^
	**N (%)**	**n (%)**	**n (%)**	**(df)**	
Scope of information					
Specific (mental health)	41 (71)	15 (63)	26 (77)	1.33 (1)	.25
Broad (health)	12 (21)	7 (29)	5 (15)	1.80 (1)	.18
Broader (general information)	5 (8)	2 (8)	3 (8)	.004 (1)	.95
Ownership structure					
Organization	36 (62)	16 (67)	20 (59)	.37 (1)	.54
Individual	10 (17)	3 (12)	7 (21)	.65 (1)	.42
Unknown	12 (21)	5 (21)	7 (20)	.001 (1)	.98
Ownership type					
Commercial	11 (19)	6 (25)	5 (15)	.97 (1)	.33
Professional	24 (41)	5 (21)	19 (56)	7.13 (1)	.008
Consumer	5 (9)	4 (17)	1 (3)	3.36 (1)	.07
Unknown	18 (31)	9 (38)	9 (27)	.80 (1)	.37
Drug company involved					
Yes	5 (9)	3 (13)	2 (6)	.78 (1)	.38
No	53 (91)	21 (88)	32 (94)		
Presence of Editorial board					
Yes	5 (9)	1 (4)	4 (12)	1.03 (1)	.31
No	53 (91)	23 (96)	30 (88)		
Health professional involved					
Yes	35 (60)	9 (38)	26 (76)	8.92 (1)	.003
No	23 (40)	15 (63)	8 (24)		
Promotion of products/services					
Yes	22 (38)	5 (21)	17 (50)	5.08 (1)	.024
No	36 (62)	19 (79)	17 (50)		
Disclaimer					
Yes	23 (40)	11 (46)	12 (35)	.65 (1)	.42
No	35 (60)	13 (54)	22 (65)		

^a^Fisher’s Exact Test.

### Accountability of the websites

Most of the websites specified the site ownership (74%, n = 43) and over half (55%, n = 32) provided sources for the content. Eleven websites mentioned the authors’ affiliations and the last date of modification. In general, accountability of the websites was poor: the mean value of the accountability score was 3.33 (SD 1.93) out of a possible maximum 9 (Table 3).

**Table 3:** Accountability scores of Finnish and Greek websites

	**Total**			**FI**			**GR**				
	**N (%)**	**Mean**	**SD**	**n (%)**	**Mean**	**SD**	**n (%)**	**Mean**	**SD**	**Chi-square (df)**	***P***^**a**^
Accountability (Range 0–9)		3.33	1.93		2.91	1.69		3.62	2.06		.18
Authors credited	21 (36)			5 (21)			16 (47)			4.19 (1)	.04
Affiliations	11 (19)			3 (27)			8 (24)			1.11 (1)	.29
Credentials	19 (33)			5 (26)			14 (41)			2.64 (1)	.10
Source given	32 (55)			15 (47)			17 (50)			.89 (1)	.35
Reference given	15 (26)			7 (47)			8 (24)			.23 (1)	.63
Site Ownership	43 (74)			16 (67)			27 (79)			1.19 (1)	.28
Site Sponsorship	20 (35)			7 (29)			13 (38)			.51 (1)	.47
Modified last 1–12 months	21 (36)			8 (33)			13 (38)			.14 (1)	.70
Last date of modification specified	11 (19)			4 (17)			7 (21)			.14 (1)	.71

^a^Fisher’s Exact Test.

When accountability of Finnish and Greek websites was compared, only one statistically significant difference was found: authorship was specified more often in Greek than Finnish websites (47% vs. 21%, *p* = .04) (Table 3).

### Interactivity of the websites

Five websites (9%) incorporated evaluation questionnaires, for example, to enable the user to provide feedback about the website or to evaluate his/her health status. About three-quarters (74%, n = 43) of all websites provided the e-mail address of the webmaster and two-third (62%, n = 36) provided an intra-site search engine. The total mean interactivity score was 1.79 (SD .87, maximum 5) which was low with no statistically significant differences between the countries (Table 4).

**Table 4:** Interactivity scores of Finnish and Greek websites

	**Total**			**FI**			**GR**				
	**N (%)**	**Mean**	**SD**	**n (%)**	**Mean**	**SD**	**n (%)**	**Mean**	**SD**	**Chi-square (df)**	***P***^**a**^
Interactivity (Range 0–5)		1.79	.87		1.17	.99		1.85	.78		.54
Intra-site search engine	36 (62)			17 (71)			19 (56)			1.34 (1)	.25
Audio/Video support	8 (14)			1 (4)			7 (21)			3.19 (1)	.07
Evaluation questionaires	5 (9)			2 (8)			3 (9)			.004 (1)	.95
Supporting Bodies	12 (21)			5 (21)			7 (21)			.001 (1)	.98
Contact webmaster	43 (74)			16 (67)			27 (79)			1.19 (1)	.28

^a^Fisher’s Exact Test.

### Aesthetics of the websites

All the websites used headings or subheadings (n = 58). Almost half of the sites did not incorporate advertisements. Two-thirds of all sites had hyperlinks to external sites, while four sites (7%) included diagrams in their content. The mean score for the total aesthetics indicator was 2.6 (SD .62, maximum 4). There were no statistically significant differences in the aesthetic ratings for the Finnish and Greek websites (Table 5).

**Table 5:** Aesthetics scores of Finnish and Greek websites

	**Total**			**FI**			**GR**				
	**N (%)**	**Mean**	**SD**	**n (%)**	**Mean**	**SD**	**n (%)**	**Mean**	**SD**	**Chi-square (df)**	***p***^**a**^
Aesthetics (Range 0–4)		2.6	.62		2.71	.55		2.53	.66		.28
Headings/subheadings	58 (100)			24 (100)			34 (100)				
Diagrams	4 (7)			1 (4)			3 (9)			.48 (1)	.49
Hyperlinks to external sites	40 (69)			18 (75)			22 (65)			.70 (1)	.40
Absence of ads	49 (85)			22 (92)			27 (79)			1.61 (1)	.20

^a^Fisher’s Exact Test.

### Content of the direct webpages

With respect to the availability of answers to five popular enquiries, the most commonly answered in Finnish direct webpages concerned diagnosis (71%), while in Greek direct webpages information about treatment (32%) was the most common. The only statistically significant difference was in the provided information about diagnosis (*p*<.001) (Table 6).

**Table 6:** Content scores between Finnish and Greek direct webpages

	**Total**			**FI**			**GR**				
**Content**	**N (%)**	**Mean**	**SD**	**n (%)**	**Mean**	**SD**	**n (%)**	**Mean**	**SD**	**Chi-square (df)**	***P***^**a**^
Diagnosis	23 (40)	.40	.49	17 (71)	.71	.46	6 (18)	.18	.39	16.6 (1)	<.001
Treatment	23 (40)	.40	.49	12 (50)	.50	.51	11 (32)	.32	.47	1.83 (1)	.18
Association	4 (7)	.07	.26	3 (13)	.13	.34	1 (3)	.03	.17	2.00 (1)	.16
Clinic	0 (0)	.00	.00	0 (0)	.00	.00	0 (0)	.00	.00		

^a^Fisher’s Exact Test.

Overall, proportionate to the potential range of scoring, the highest scoring indicator was aesthetics of the websites (2.6 out of 4), followed by accountability (3.33 out of 9) and interactivity (1.79 out of 5).

## Discussion

This study was designed to assess, describe and compare Finnish and Greek websites appearing first on ‘Google’ when using a search term on schizophrenia or related conditions. The websites were analysed with respect to type and characteristics, accountability, interactivity, aesthetics and content of the websites. It seems that first-appearing Finnish and Greek websites, providing mental health-related information were of low quality (reflecting the fact that few of the Silberg criteria, on average, were satisfied) with respect to the assessed indicators. These results are similar to those reported in Reavley and Jorm’s review [38] of 23 studies of the quality of mental disorder information websites. The current findings are valuable because there is evidence that people with schizophrenia perceive the Internet as an important and influential source of information [19]. Although research interest in the quality of online health information has been apparent for at least 17 years [59,60], little seems to have changed regarding the development and dissemination of online health information.

Although compared to Greek citizens, Finnish citizens are much more likely to seek health information online [61], and to have greater access to the internet [29], and more advanced ICT skills [28,30], our study found no evidence that the first-returned Finnish websites providing schizophrenia-related information were of better quality than Greek websites. This finding does not exclude the possibility that there are high quality Finnish and Greek mental health websites, but it does demonstrate that if high quality sites exist they are not among the first 20 results when a schizophrenia-related Google search is performed.

In the present analysis, accountability of Greek websites was higher than the Finnish ones. This could be due to the profusion of private doctors and private diagnostic clinics in Greece [62], who use websites to advertise their professional profile and attract consumers. This is supported by the finding that service promotion was more common on the Greek websites than on Finnish websites. In contrast to their Finnish counterparts, most Greek consumers tend to pay privately for their healthcare [63]. The latter may also explain the significantly greater level of health professional ownership and professional content development of mental-health related websites in Greece compared to Finland. Identification of the ownership structure of the Finnish websites was unclear. There were no significant differences between the Finnish and Greek websites with respect to other characteristics of websites such as scope, country and whether a drug company was involved.

Website interactivity was poor in both countries. Interactivity is considered an important website element offering a way to communicate with users and engage them in using the provided services [64]. It is unclear if the present findings reflect the poor interactivity of websites, in general, or if they reflect a more specific limitation of mental health websites in a field where mental health consumers are often inaccurately perceived as passive users of health services [65]. Providing consumers with trustworthy online health information has the potential to lead to better outcomes when given information and choices [65,66]. Our findings also suggest that the Finnish and Greek websites are primarily designed for passive information delivery, rather than providing interactive tools and support. It is critical to consider consumers’ needs and opinions [24], if the Internet is to be an effective psychoeducation tool [14]. Moreover, supporting the inclusion of vulnerable populations, like people with schizophrenia, into an information society is emphasized by the European Commission [67].

Our analysis demonstrated that the content of the webpages deriving directly from the first 20 ‘Google’ results, and in particular whether they provided answers to common consumer enquiries, was low. It has been reported [25,54,68] that online health-seekers search for information related to a disease, symptoms, treatment options, clinics and patients’ associations. In the current study it was found that this information was typically not available within the first webpage of the included websites.

In our study we included ‘wikis’ in the analysis, although their use as a reliable health resource has been criticized [69,70]. This decision was made on the grounds that web search engines often lead users to Wikipedia [71]. Wikis are favored online sources among consumers with schizophrenia [19]. Large amounts of health information are provided in Wikipedia [69], and there has been some evidence that health professionals believe that popular Wikipedia articles (including those on ‘schizophrenia’) are of ‘good quality’ [70]. Such evaluations are typically focused on the accuracy of the content rather than on the interactivity or aesthetics of the site. While accurate content is necessary [45], it is also crucial to ensure that information is delivered in a way that a person with schizophrenia or a related condition can become informed, since symptoms of these disorders, such as attention deficit or delusional misinterpretations, may compromise Internet use [19]. We attempted to assess all generated websites such as wikis, blogs, forums, etc., regardless of their structure, in order to focus on those websites that are returned in typical online searches for mental health information. However, future research should consider investigating comparative quality across these different types of resources including wikis.

### Limitations

There are a number of limitations of this study. First, the online search was performed in November 2011 and considering the changing nature of the Internet, it is unsure that the results of this study will remain the same in the future. Second, there are no studies on how Finnish and Greek people diagnosed with schizophrenia retrieve online mental health information. Hence, they may use a search engine other than ‘Google’ or use different search terms or methods to acquire online health information about their illness. In addition, the conveniece sampling method used for the selection of five webpages for each website, does not ensure that someone could access and read the specific five webpages. Third, the information gathered about Finnish and Greek websites used a limited range of specific quality measures, excluding, for example, readability [26] and quality of content [45,56,72]. In Finland, official clinical guidelines for mental disorders are available, but not in Greece. For this reason, the current study did not employ a measure of the quality of content based on evidence-based guidelines. Fourth, the quality indicators of this study could potentially consist of more items. For example, the aesthetics indicator did not measure the presence or not of images or video or other media (only diagrams) in our included webpages, although such material has the potential to assist people to better understand the text they read [73]. Fifth, the availability of answers in common inquiries (diagnosis, treatment, associations, clinics) was assessed from the direct webpages. Therefore, the content description is limited to one webpage only and, thus, does not apply to the content of the whole website. Additionally, only the presence or absence of specific content was assessed (diagnosis, treatment, patient associations, clinics), without evaluating the accuracy or comprehensiveness of the provided information. Thus, even when the webpage achieved a positive rating on the current scoring system, the content of the webpage might have been of low evidence-based quality. Last, inter-rater reliability was only calculated for the content of webpages, since it was the only indicator assessed by two same-language raters. However, the coding of the other indicators was discussed for all data, between the Finnish and the Greek rater.

## Conclusions

All assessed indicators of Finnish and Greek websites, appearing first on ‘Google’ when using a schizophrenia-related search term achieved low scores. This is despite the difference in ICT context in the two countries and in particular the greater Internet use and access, and ICT experience in Finland. Regardless of the latter disparities between Finland and Greece, those with schizophrenia and related conditions receive a similar level of online experience when they spontaneously search for schizophrenia-related information via ‘Google’.

### Implications

This study provides a foundation for the future development of websites on the topic of schizophrenia, and suggests that improvement in many aspects of website quality is needed. Although the quality of mental health information websites may have improved over the past decade [38], the current findings suggest the need for increased awareness about the various quality indicators among website developers, bloggers, ‘wiki’, forum contributors, and others who upload schizophrenia-related content online.

All developers, including health professionals who are significant online health information contributors [70] should be encouraged to comply with international standards and guidelines before uploading health-related information. Such instruments provide an essential tool to guide developers to produce usable websites including those intended for people with schizophrenia. This could promote homogeneity, easy access, and clear interpretation of the online health information, and thereby, support and educate consumers and their families. Additionally, health professionals should be open to provide guidance on which online health information can be trusted and why, since people diagnosed with a mental disorder are also searching for health information online [27].

Furthermore, the most popular search engines could facilitate the delivery of high quality websites. For example, a search engine might potentially be programmed to return high quality information websites [35,74] prior to sponsored results and lesser quality websites. Moreover, Google’s country specific versions might follow Google USA’s example [75], where they partnered with the National Institute of Health to generate relevant health-related information in response to consumers’ searches [76].

## Abbreviations

ICT: Information and communication technologies.

## Competing interests

The authors declare that they have no competing interests.

## Authors’ contributions

CA led the conception and design, acquisition of data, analysis and interpretation of data, drafted and revised the manuscript and gave final approval of the version to be published. HH made substantial contributions to analysis and interpretation of data, was involved in drafting the manuscript or revising it critically for important intellectual content and gave final approval of the version to be published. SS made substantial contributions to acquisition and analysis of data and gave final approval of the version to be published. CL made substantial contributions to conception, interpretation of data, was involved in drafting the manuscript or revising it critically for important intellectual content and gave final approval of the version to be published. KG made substantial contributions to conception, interpretation of data, was involved in drafting the manuscript or revising it critically for important intellectual content, and gave final approval of the version to be published. MV made substantial contributions to conception and design, analysis and interpretation of data, and drafted the manuscript as well as revising it critically for important intellectual content. MV also gave final approval of the version to be published. All authors read and approved the final manuscript.

## Pre-publication history

The pre-publication history for this paper can be accessed here:

http://www.biomedcentral.com/1472-6947/13/98/prepub
